# Progressive coronary aneurysms in Kawasaki disease: A case report and long-term follow-up

**DOI:** 10.1016/j.radcr.2025.01.090

**Published:** 2025-03-08

**Authors:** Thomas Saliba, Franck Nevesny, Panagiotis Antiochos, David Rotzinger

**Affiliations:** a Radiodiagnostic department, Centre Hospitalier Universitaire Vaudois, Rue du Bugnon 46, 1005 Lausanne, Switzerland; b Cardiology department, Centre Hospitalier Universitaire Vaudois, Rue du Bugnon 46, 1005 Lausanne, Switzerland

**Keywords:** Kawasaki, Aneurysm, Von Willebrand, Thrombosis, Complication, Treatment

## Abstract

Kawasaki disease (KD) is a vasculitis that primarily affects children under 5 years of age with coronary artery aneurysms being a significant complication that can lead to long-term cardiovascular risks such as thrombosis and stenosis. We report the case of a 13-year-old boy who was diagnosed with KD after presenting with fever, skin lesions, and conjunctivitis. Cardiac imaging revealed aneurysms of the intraventricular artery, the circumflex artery, and the right coronary artery. Despite initial treatment with intravenous immunoglobulin, aspirin, low molecular weight heparin, and subsequent acenocoumarol therapy, the patient experienced progressive thrombosis of the intraventricular artery aneurysm over 3 years. Investigations revealed a hypercoagulable state due to hyperactive Von Willebrand factor, prompting treatment adjustments that resulted in gradual improvement of the thrombosis. This case underscores the critical need for early imaging, timely diagnosis, and long-term monitoring of KD patients, as coronary aneurysms may persist or progress despite prompt therapy. Rigorous follow-up, tailored anticoagulation strategies, and regular imaging are essential to minimize the risk of life-threatening cardiovascular complications.

## Introduction

Kawasaki disease was first described in 1967 by Dr. Tomisaku Kawasaki [1]. It is an acute vasculitis of unknown origin that primarily affects children under the age of 5 [2]. The disease has a higher incidence in ethnically Asian children, with Japan reporting the highest rate at 360 per 100 000, followed by Korea [2]. In contrast, the incidence for ethnically Caucasian children is 7 per 100,000 [3]. There is a male preponderance with a sex ratio of 1.5:1 [3].

Though the exact aetiology is unknown, some authors suggest that the hygiene hypothesis may be a contributing factor [3]. A large Japanese study reported cardiac complications in 7.9% of cases, as well as 5.6% of patients having coronary dilations, 0.82% having coronary aneurysms and 0.13% developing giant aneurysms [2].

Without treatment, approximately 25% of patients will develop a coronary artery aneurysm [4]. Even with prompt immunoglobulin and aspirin treatment, 3%-5% of patients will still develop coronary aneurysms [4].

### Case report

A 13-year-old boy with a 5-day history of fever, skin lesions, cheilitis, a strawberry tongue, cervical lymphadenopathy, and bilateral conjunctivitis was seen by his paediatrician. Suspecting scarlet fever, the paediatrician initially prescribed azithromycin. When the symptoms failed to resolve after 5 days, penicillin was added to the treatment regimen.

On the tenth day, the patient presented to the emergency department, where the Kawasaki disease was diagnosed based on the criteria of the American Heart Association (AHA). This included a 5-day history of fever and fulfilling 4 out of 5 major diagnostic criteria: bilateral conjunctivitis, cheilitis, rash and lymphadenopathy.

A heart ultrasound revealed aneurysms of the right coronary (RCA) and left anterior descending (LAD). Immediate treatment with intravenous immunoglobulins was initiated, alongside 100 mg/day of aspirin and low molecular weight heparin to prevent thrombosis. The fever subsided after 2 days. Two days later, acenocoumarol was added to the treatment regimen, and heparin was discontinued on the 8th day after admission at which time the patient was discharged with a treatment regimen of aspirin and acenocoumarol.

**Fig. 1:**
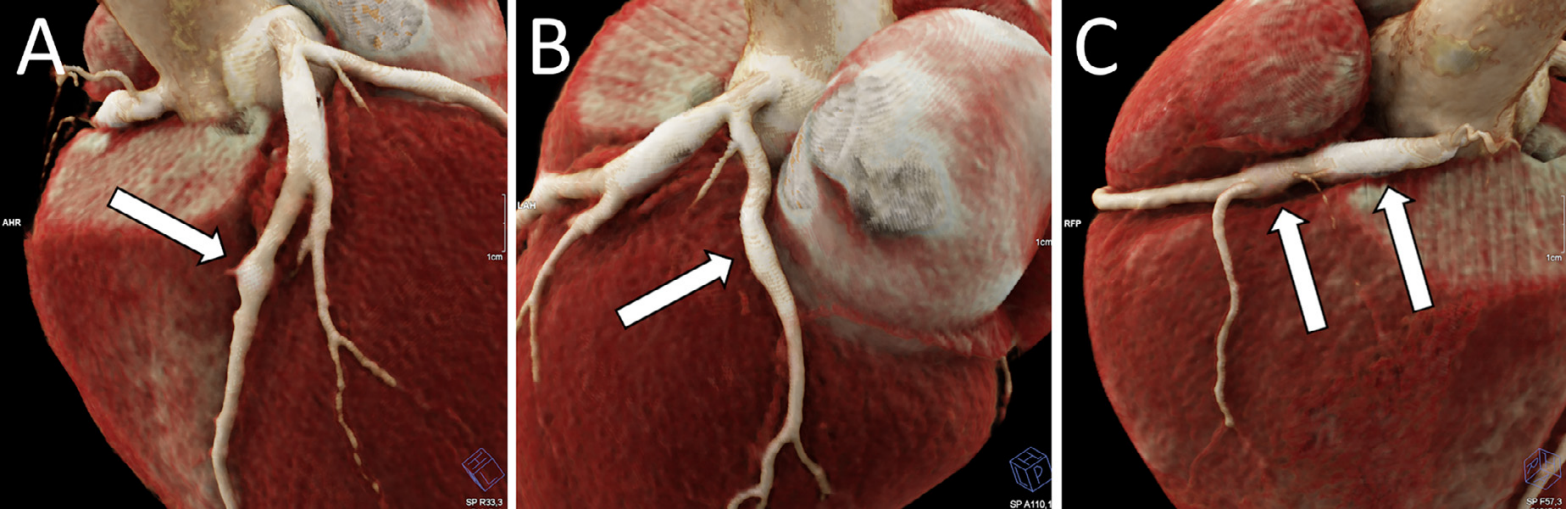
Three-dimensional-volume rendering of a coronary CT-scan showing multiple coronary aneurysms. There are aneurysms of the intraventricular artery (A, arrow), the circumflex artery (B, arrow), and right coronary artery (C, arrow).

**Fig. 2:**
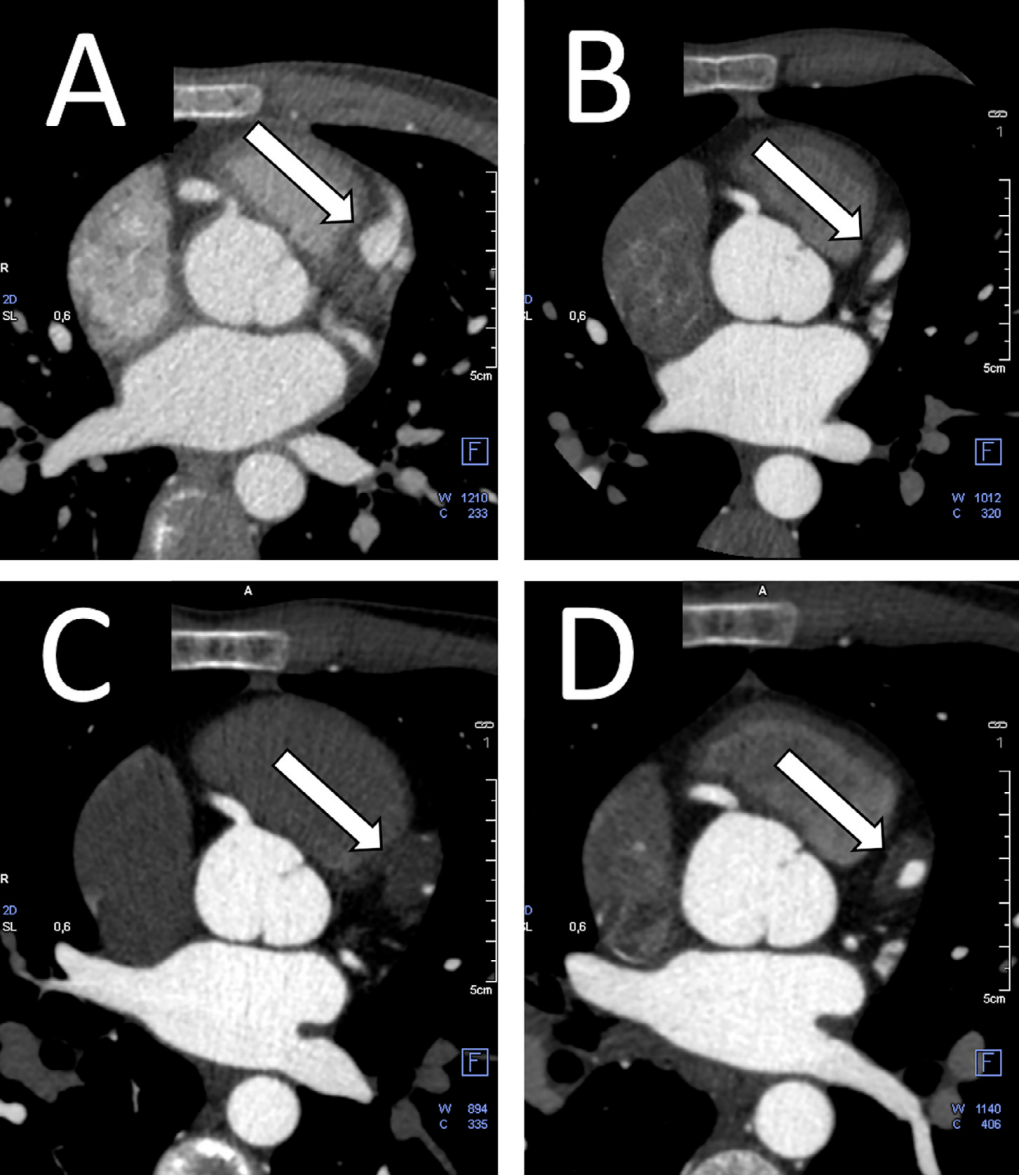
Contrast-enhanced coronary CT showing the progressive thrombosis of the aneurysmal intraventricular artery (A), followed by a thrombosis and decrease of lumen size (B), near occlusion on a follow-up exam 3 years after initial presentation (C), and subsequent improvement (D).

Three months after discharge, a follow-up coronary CT scan revealed persistent aneurysms (Fig. 1, Fig. 2, panel A). It was agreed that the patient would return for a CT scan the following year. However, the patient presented 2 years later, at which point a coronary CT scan showed a partial thrombosis of the LAD aneurysm along with a fusiform aneurysm of the left circumflex artery and the RCA. The RCA aneurysm had a mural thrombosis, causing a 50% stenosis (Fig. 2, panel B). Further investigation revealed that the patient had been taking an insufficient dose of acenocoumarol, with consistently low INR values (<2). Subsequent testing identified hyperactivity of the patient's Von Willebrand factor, contributing to a hypercoagulable state. The patient's treatment regimen was adjusted accordingly, and a follow-up CT-scan was scheduled for the following year.

Three years after the initial diagnosis, coronary CT revealed progression of the LAD thrombosis, with increased size and further vessel occlusion (Fig. 2, panel C). The patient's treatment was adjusted again, and a follow-up CT scan was scheduled 6 months later. The subsequent CT scan showed a slight regression of the thrombosis, indicating effective treatment (Fig. 2, panel D).

**Fig. 3:**
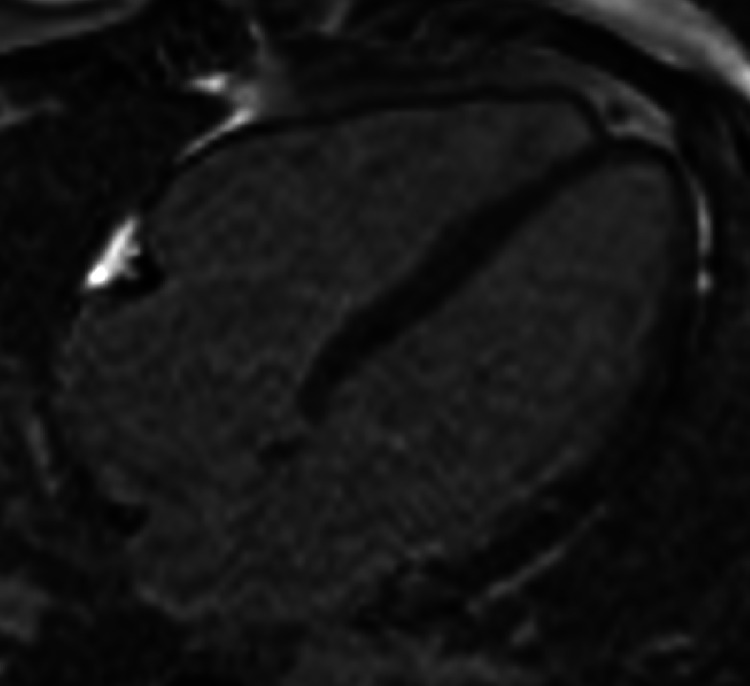
1.5 T MRI postcontrast T1 weighted sequence showing the absence of subendocardial late gadolinium enhancement and thus no signs of myocardial infarcts.

An MRI performed later showed no signs of ischemia, likely due to the development of collateral circulation as the thrombosis progressed (Fig. 3).

The patient is currently undergoing regular follow-ups, and his condition continues to improve.

The patient provided informed consent for the publication of this paper.

## Discussion

Kawasaki disease is an inflammatory vasculitis of medium-sized vessels and is characterized by persistent [2]. Diagnosing the disease is challenging due to the lack of pathognomonic features, requiring reliance on a set of clinical criteria [2]. The disease has a particular predilection for coronary arteries, with aneurysm formation occurring in approximately a quarter of untreated patients [2]. When coronary aneurysms develop, they typically affect the proximal segments of the main arterial branches [5].

The pathophysiology involves self-limiting artery necrosis that progresses into fusiform aneurysms, chronic vasculitis and eventually stenosis and thrombosis. These processes are thought to be linked to luminal myofibroblast proliferation [2]. Cardiac complications can result in myocardial infarction or even sudden death, either in the acute phase or in adulthood [2]. However, in the absence of myocardial ischemia, stenosis or even large aneurysms can remain asymptomatic [5]. Notably, up to 5% of acute coronary syndromes in patients under 40 years of age are attributable to Kawasaki disease, underscoring the critical need for long-term follow-up and monitoring [5].

The first line of treatment is a single, high dose of intravenous immunoglobulin, combined with aspirin [2]. Echocardiography serves as the primary imaging modality and should be performed as early as possible to evaluate the main coronary arteries, facilitating timely diagnosis and treatment. One study reported that 81% of coronary abnormalities were detected on the initial echocardiography [2]. During the early phase, coronary dilatation is defined as a diameter exceeding 3 mm in children under 5 years and 4 mm in those over 5 years, or as a lesion that is more than 1.5 times the diameter of an adjacent normal segment [2,3]. Aneurysms can be further subclassified into small, medium, or giant if the vessel equals or exceeds 3 mm, 4 mm or 8 mm, respectively [2].

To account for variability in patient size, the American Heart Association has established adjusted z-scores, which help predict patient outcomes and guide the frequency of follow-up examinations [2,3]. Among patients with coronary artery aneurysms, complications are rare, with only 1% of events occurring in those with a z-score of less than 10 [4].

Although aspirin, warfarin and low molecular weight heparin are commonly used for thrombosis prophylaxis, coronary thrombosis can still occur in patients who develop aneurysms. One potential complication is embolization, in which fragments of the thrombus occlude downstream vessels [4]. Prompt detection and treatment are essential to minimize damage [4].

Kawasaki disease is rare in adults, accounting for only 0.7% of cases. However, isolated adult cases have been reported, including one involving a 20-year-old woman who developed the disease after contracting SARS-CoV-2 [3,6].

## Conclusion

Kawasaki disease predominantly affects young children, with coronary artery aneurysms being a common complication. Patients who develop aneurysms require ongoing follow-up to monitor for thrombosis, which can lead to ischemic heart disease. We presented the case of a 14-year-old boy who developed Kawasaki disease, complicated by the formation of a giant coronary aneurysm. This case highlights the importance of investigating potential factors that may contribute to a hypercoagulable state in patients with Kawasaki disease and underscores the critical need for rigorous follow-up to promptly identify and manage complications.

## Patient consent

We hereby declare that we the patient provided informed consent that their data could be used for scientific purposes.
